# Multifunctional MoS_2_@g-C_3_N_4_ Nanocomposites for Dual-Mechanism Removal of Drug Molecules and Azo Dyes

**DOI:** 10.1021/acsomega.4c09040

**Published:** 2025-03-20

**Authors:** Salsabil Marouch, Gokhan Sarp, Mustafa Soylak, Erkan Yilmaz

**Affiliations:** † Laboratory of Chemistry and Environmental Chemistry (LCCE), Department of Chemistry, Faculty of Matter Sciences, Batna-1 University, Batna 05000, Algeria; ‡ Department of Chemistry, Faculty of Science, Erciyes University, Kayseri 38039, Turkey; § ERNAM−Nanotechnology Application and Research Center, Erciyes University, Kayseri 38039, Turkey; ∥ Department of Analytical Chemistry, Faculty of Pharmacy, Erciyes University, Kayseri 38039, Turkey; ⊥ Technology Research and Application Center (TAUM), Erciyes University, Kayseri 38039, Turkey; # Erciyes Teknopark-ChemicaMed Chemical Inc., Erciyes University Technology Development Zone, Kayseri 38039, Turkey

## Abstract

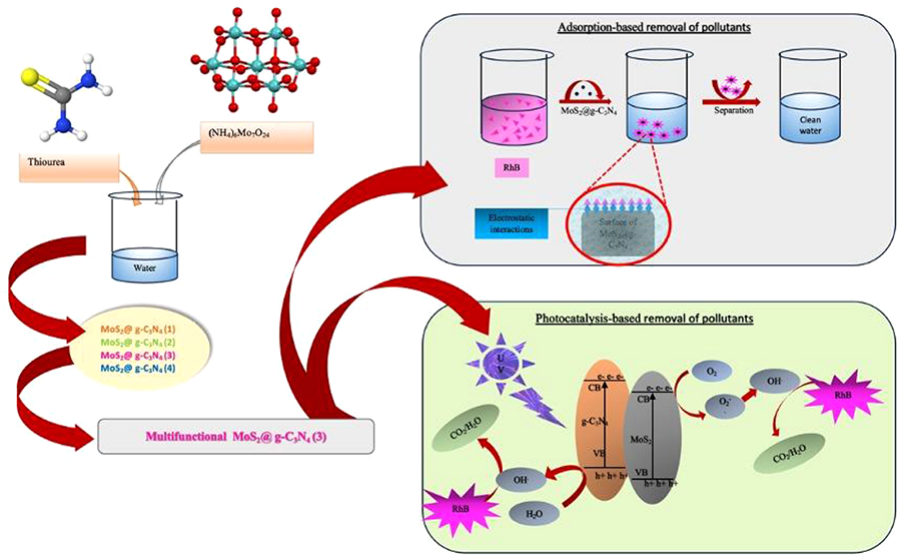

This work examined the simple synthesis of a multifunctional nanomaterial based on MoS_2_ and g-C_3_N_4_ nanosheets (NSs) combination for the dual, adsorption-based and photocatalytic degradation-based removal of Rhodamine B (RhB), sildenafil citrate (SLD), and fluoxetine (FLX) from water. The study intended to identify the best ratio of MoS_2_ to g-C_3_N_4_ to obtain the best adsorption and photocatalytic performances; therefore, the MoS_2_@g-C_3_N_4_ nanocomposites were synthesized with four different ratios of MoS_2_ NSs and g-C_3_N_4_ NSs, then characterized with FT-IR, XRD, and SEM techniques. Consequently, MoS_2_ to g-C_3_N_4_ (3) was identified to be the most effective nanomaterial with outstanding adsorption and photocatalyst abilities. The specifically optimized nanocomposite was further experimented with for SLD and FLX removal, demonstrating high efficiency regarding all pollutants with the highest adsorption percentage at pH 4.0 for RhB, pH 8.0 for SLD, and pH 9.0 for FLX, respectively. A higher photocatalytic degradation rate was realized under UV light with complete decolorization of RhB in 300 min and SLD in 210 min. Thus, the outstanding adsorption and photocatalytic ability of the MoS_2_@g-C_3_N_4_ (3) nanocomposite material point toward the fact that it may be used to treat a wide range of environmental pollutants.

## 1 Introduction

Industries and urban development over the decades have enormously enhanced the release of several organic pollutants to water bodies.^1^ Such compounds include dyes, pharmaceuticals, and personal care products that are cytotoxic, persistent, and bioaccumulative; hence, they are hazardous to the environment and human health. Among these contaminants, Rhodamine B (RhB), Sildenafil citrate (SLD), and Fluoxetine (FLX) are particularly concerning due to their widespread and extensive usage and high persistence in nature,^2−4^ Rhodamine B, commonly used in industries, is carcinogenic, mutagenic, and teratogenic, with the potential to disrupt ecosystems and harm human health,^5−7^ Sildenafil, often found in water bodies due to improper disposal, affects aquatic organisms by disrupting their endocrine systems and reproductive functions.^8^ Fluoxetine, an antidepressant, alters aquatic species’ behavior, feeding habits, and reproduction, even at trace levels. These risks underline the need for effective strategies to prevent water contamination and safeguard ecosystems and public health.^9−11^

Many water treatment technologies have been developed to address the challenges posed by these pollutants, including biological processes, chemical oxidation, chlorination, and advanced oxidation processes (AOPs) like photolysis, photocatalysis, and ozonation. Additionally, methods like solar desalination and membrane filtration, including microfiltration, ultrafiltration, nanofiltration, and reverse osmosis, showed high effectiveness and selectivity for removing many pollutants. Among these, adsorption and photocatalytic degradation stand out for their high efficiency and unique ways of treating contaminants.^12−15^ Among these advanced methods, adsorption and photocatalytic degradation stand out for their effectiveness and innovation. The adsorption process relies on surface interactions where functional groups on an adsorbent selectively attract and capture dissolved contaminants through forces such as van der Waals and electrostatic interactions. Since contaminants remain chemically unchanged, they can be released via desorption for reuse or further treatment.^16^ Conversely, photocatalytic degradation employs semiconductor photocatalysts to harness photon energy and initiate redox reactions, generating reactive oxidative species (ROS). These highly reactive species decompose harmful substances into less hazardous products like carbon dioxide and water, making photocatalysis a powerful tool for breaking down persistent pollutants.^17−19^

The evolution of multifunctional nanomaterials represents a promising feature in this field. Due to their unique properties, they can be used in both processes, adsorption and photocatalysis. Playing the role of adsorbent in the adsorption-based removal, their high surface area and the functional groups on their surface provide more active sites for pollutants to adhere to, resulting in an effective purification process. In photocatalysis, these nanomaterials are used as photocatalysts, facilitating the promotion, effective generation, and activity of radical oxidative species, which enhances the degradation of pollutants.^20^

This manuscript aims to analyze the possibility of using a combination of MoS_2_ NSs and g-C_3_N_4_ NSs as a new multifunctional material for removing RhB, SLD, and FLX by the dual mechanism. MoS_2_ is an inorganic compound with one molybdenum atom and two sulfur atoms. It is considered part of transition metal dichalcogenides (TMDs) with high adsorption capacity and photocatalytic activity.^21,22^ At the same time, the g-C_3_N_4_ N NSs are a metal-free nanomaterial that exhibits a layered structure similar to graphite. The C_3_N_4_ NSs can use light to break down pollutants and are chemically stable.^23,24^ These combined properties make them effective for removing contaminants and a good option for sustainable water purification technologies.

More precisely, this work aims to identify the most suitable MoS_2_ NSs to g- g-C_3_N_4_ NSs ratio effective for RhB dye removal using adsorption and photocatalytic procedures. In addition, the performance of the optimized nanomaterial will be tested to remove other pollutants, such as SLD and FLX, which will showcase its flexibility and potential to tackle different environmental contaminants in water treatment.

## 2 Experimental Section

### 2.1 Chemicals and Reagents

Thiourea (H_2_NCSNH_2_), ammonium heptamolybdate ((NH_4_)_6_Mo_7_O_24_), fluoxetine, Sildenafil citrate, and RhB were all purchased from Merck. Deionized water (resistivity 18.2 MΩ·cm) was obtained using the Milli-Q deionized water system (Millipore, USA). All chemicals and solvents were used as received without further purification.

### 2.2 Synthesis of MoS_2_@g-C_3_N_4_ Nanocomposite

MoS_2_@g-C_3_N_4_ nanocomposites materials were synthesized according to the method of Xue et al.,^25^ where thiourea and ammonium molybdate were dissolved in 100 deionized water with varying amounts of ammonium molybdate, as shown in Table 1. Each mixture was left in an oven at 100 °C until the water evaporated (12 h). Then, the resulting solid was placed in a tube furnace under argon gas flow and left at 550 °C until the reaction was completed. The obtained MoS_2_@g-C_3_N_4_ nanocomposite was washed with water and ethanol and dried in a vacuum oven at 50 °C for 16 h.

**Table 1:** Ratios of Thiourea and Ammonium Molybdate Used in the Synthesis of MoS_2_@g-C_3_N_4_ Nanocomposite

	**(NH**_**4**_**)**_**6**_**Mo**_**7**_**O**_**24**_		**thiourea**		**molar ratio**
nanocomposite	(g)	(mol)	(g)	(mol)	(thiourea: ammonium molybdate)
g-C_3_N_4_	0	0	10	0.1314	1:0
MoS_2_@g-C_3_N_4_ (1)	0.003	2.43 × 10 ^–6^	10	0.1314	54,198:1
MoS_2_@g-C_3_N_4_ (2)	0.03	2.43 × 10 ^–5^	10	0.1314	5408:1
MoS_2_@g-C_3_N_4_ (3)	0.3	2.43 × 10 ^–4^	10	0.1314	540:1
MoS_2_@g-C_3_N_4_ (4)	3	2.43 × 10 ^–3^	10	0.1314	54:1

### 2.3 Instruments

Fourier Transform Infrared Spectroscopy (FT-IR) characterized the chemical composition of the synthesized nanomaterials using a Thermo Scientific Nicolet 6700 spectrometer with the attenuated total reflectance (ATR) method. The crystallographic structure was analyzed using a Bruker AXS D8 X-ray powder diffractometer with Cu Kα radiation (λ = 0.15406 nm). The scan range (2θ) was from 5° to 90°. The morphology of the synthesized nanomaterials was studied using a Zeiss-Gemini 500 Field Emission Scanning Electron Microscope (FE-SEM) at an operating voltage of 15 kV. The surface area, pore volume, and pore width of the synthesized MoS_2_@g-C_3_N_4_ (1) and MoS_2_@g-C_3_N_4_ (3) nanocomposites were performed by BET-N_2_ method using a Micromeritics Gemini VII BET analyzer. The ζ-potentials of MoS_2_@g-C_3_N_4_ (3) nanocomposite were measured at different pH values using a Malvern ZEN2600 Zetasizer Nano ZS.

The analysis of SLD and FLX was conducted using HPLC-DAD. For SLD, isocratic conditions were employed with a mobile phase composed of a 40:60 (v/v) mixture of pH 6.5 phosphate buffer solution and methanol. The stationary phase was C18 (10 × 4.5 mm, 5 μm), supplied by USEM Research and Development Company (Kayseri, Turkey). Detection was carried out at a wavelength of 292 nm. For FLX, the analysis also utilized isocratic conditions. The chromatographic conditions were adapted from the technique developed by Baker et al.,^26^ with some modifications. The mobile phase consisted of pH 3.0 phosphate buffer, acetonitrile, and methanol in a volumetric ratio of 60:33:7. The stationary phase was C18 (10 × 4.5 mm, 5 μm), also provided by USEM Research and Development Company (Kayseri, Turkey).

### 2.4 Evaluation of the Performance of the Synthesized Nanomaterials

The performance of the synthesized MoS_2_@g-C_3_N_4_ nanocomposites was evaluated by testing their effectiveness in removing RhB through adsorption and photocatalytic degradation.

#### 2.4.1 Photocatalysis Degradation-Based Removal of Contaminants

In the photocatalytic experiments, 20 mg·L^–1^ of RhB, SLD or FLX solution was prepared in 150 mL of deionized water. To this solution, 25 mg of the synthesized MoS_2_@g-C_3_N_4_ nanocomposite was added. The mixture was stirred in the dark on a magnetic stirrer for 12 h to ensure complete adsorption. Before exposing the mixture to UV light, a 1.0 mL sample was taken from the solution. The remaining solution was placed under a 400 W UV lamp, approximately 15 cm above the solution’s surface. The degradation of RhB, SLD or FLX was monitored by taking 1.0 mL samples at 30 min intervals over 300 min. While the concentration changes of RhB in the solution were determined using a UV–vis spectrophotometer at 556 nm, the concentration changes for SLD and FLX were monitored by using Ultra Performance Liquid Chromatography with Diode-Array Detection (UPLC-DAD) instrument. The calibration line method for UV–vis spectrophotometry and UPLC-DAD methods were used to determine the concentrations of RhB, SLD and FLX molecules.

The following equation (eq 1) gives the photodegradation percentage.
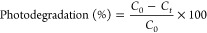
1

where *C*_0_ (mg·L^–1^) and *C*_*t*_ (mg·L^–1^) represent the concentration of RhB at the initial time and time *t*, respectively.

RhB’s photocatalytic-based removal rate and synthesized materials’ kinetics are explored using zero-order, pseudo-first-order, and pseudo-second-order kinetic models (eqs 2 –4).^27^
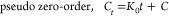
2
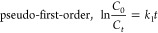
3
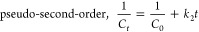
4

where *C*_0_ and *C*_t_ are the concentrations of RhB at *t* = 0 and at time *t*, respectively (mg·L^–1^), t is the contact time in hours, and *k*_0_, *k*_1_, and *k*_2_ are zero-order, first-order, and second-order rate constants, respectively.

The reusability of the optimized material as a photocatalyst was investigated by repeating the photocatalysis-based removal experiment steps five times.

#### 2.4.2 Adsorption-Based Removal of Contaminants

Adsorption-based removal experiments for RhB, SLD and FLX were conducted on sample solutions including 10 mg·L^–1^ of RhB or SLD or 5 mg·L^–1^ of FLX with the addition of appropriate buffer solutions. To these solutions, 25 mg of MoS_2_@g-C_3_N_4_ nanocomposite was added. The tubes were then transferred to a vortex device and stirred for 10 min to ensure thorough mixing. This was followed by a 10 min treatment with ultrasonic waves to enhance the adsorption process. Subsequently, the mixtures underwent another 10 min vortexing session to ensure complete interaction between the MoS_2_@g-C_3_N_4_ nanocomposite and the target molecules. After the adsorption process, centrifugation gently separated the loaded adsorbent from the solution. A precise volume of 1.0 mL was extracted from the resulting solution to determine the nonadsorbed target molecule concentration. As the RhB concentration in each sample was measured using UV–vis spectrophotometry at a wavelength of 556 nm, the concentration of SLD and FLX was determined with Ultra Performance Liquid Chromatography with Diode-Array Detection (UPLC-DAD) instrument.

The adsorption percentage is given by the following equation (eq 5)^28^
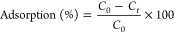
5

where *C*_0_ (mg·L^–1^) and *C_t_* (mg·L^–1^) are the RhB concentrations at the initial time and time *t*, respectively.

The optimized material’s adsorption capacity and isotherm at pH 4.0 were studied by immersing 25 mg of MoS_2_@g-C_3_N_4_ nanocomposite in 10 mL of RhB solution with various initial concentrations ranging from 1.0 to 60 mg·L^–1^.

The adsorption capacity (*Q_t_*) of the material was determined using the following equation (eq 6):^29^
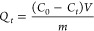
6

where *Q*_*t*_ (mg·g^–1^) is the adsorption capacity at *t* time, *C*_0_ (mg·L^–1^) and *C_t_* (mg·L^–1^) are the initial concentration and concentration at *t* time of RhB, respectively, *V* (L) is the volume of the RhB solution, and *m* (g) is the mass of the adsorbent.

To study the nature of the interactions between the adsorbent and RhB, the experimental data were analyzed using the Langmuir and Freundlich isotherm models (eqs 7 and 8).^30^

The Langmuir isotherm was applied to the data, and the linear form (eq 7) of the equation was used:
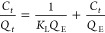
7

where *C_t_* is the equilibrium concentration of RhB (mg/L), *Q_t_* is the amount of RhB adsorbed per unit mass of adsorbent at the equilibrium(mg/g), *Q*_E_ is the maximum adsorption capacity (mg/g), and *K*_L_ is the Langmuir constant related to the affinity of the binding sites (L·mg^–1^).

The Freundlich isotherm was applied using the linear form of the equation (eq 8):
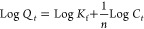
8

where *Q_t_* is the amount of RhB adsorbed per unit mass of adsorbent at the equilibrium (mg·g^–1^), *C_t_* is the equilibrium concentration of RhB (mg·L^–1^), *K*_f_ is the Freundlich constant indicative of the adsorption capacity (mg·g^–1^), and 1/*n* is the adsorption intensity.

## 3 Results and Discussion

### 3.1 Characterization of the Synthesized Nanomaterials

**Figure 1:**
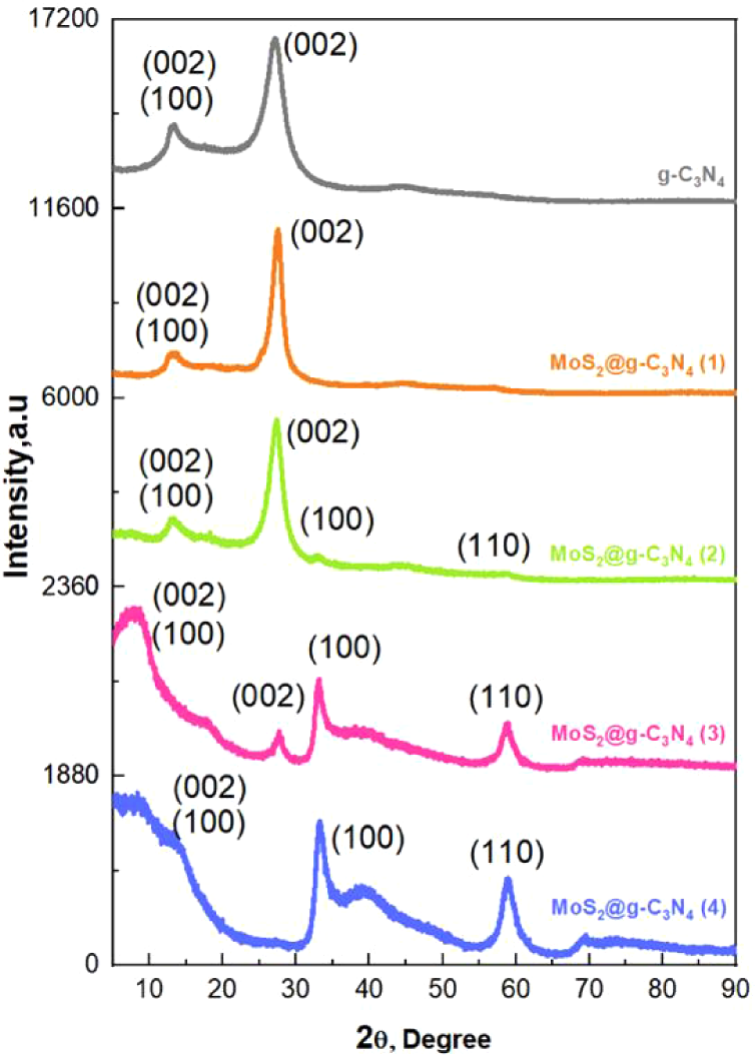
XRD spectra of the synthesized MoS_2_@g-C_3_N_4_ nanocomposites.

The results of the XRD analysis for g-C_3_N_4_ and MoS_2_@g-C_3_N_4_ nanocomposites, presented in Figure 1, reveal key structural features and interactions between the components. The characteristic diffraction peaks of g-C_3_N_4_ were identified at 12.8° corresponding to the (100) plane and 27.6° corresponding to the (002) plane. In contrast, MoS_2_ exhibited peaks at 13.4° (002), 33.1° (100), and 58.7° (105), aligning with its hexagonal crystal structure.^25,31−33^ The diffraction peaks for both g-C_3_N_4_ and MoS_2_ were evident across the composites, with their intensity and width varying with MoS_2_ content, indicating changes in crystallinity and interactions. At low MoS_2_ content (MoS_2_@g-C_3_N_4_ (1)), the XRD pattern closely resembled pure g-C_3_N_4_, suggesting minimal structural disruption. For MoS_2_@g-C_3_N_4_ (2), a slight broadening of the g-C_3_N_4_ (002) peak was observed, implying initial interactions between the two components. Significant changes were noted in MoS_2_@g-C_3_N_4_ (3), with a broad peak appearing at 8°, attributed to expanded MoS_2_ interlayer spacing caused by g-C_3_N_4_ intercalation and reduced intensity of the g-C_3_N_4_ peak at 27.6°, indicating disrupted stacking.^33^ In MoS_2_@g-C_3_N_4_ (4), MoS_2_-related peaks became more pronounced, and the g-C_3_N_4_ peak intensity further decreased, reflecting extensive structural reorganization. The average crystallite size and strain calculations using the Scherrer and Williamson-Smallman equations have been performed to confirm the structural changes.^34^ The results showed that the crystallite size increases with increasing MoS_2_ content: 1.84 nm for pure g-C_3_N_4_ to 2.11 nm for MoS_2_@g-C_3_N_4_ (1), 2.3 nm for MoS_2_@g-C_3_N_4_ (2), 4.26 for MoS_2_@g-C_3_N_4_ (3), and 6.92 nm for MoS_2_@g-C_3_N_4_ (4). In contrast, the strain exhibits a nonlinear behavior, starting at 7.37% for pure g-C_3_N_4_, rising to 11.22% for MoS_2_@g-C_3_N_4_ (1), then decreasing to 8.86%, 6.80%, and finally 0.07% for MoS_2_@g-C_3_N_4_ (2), (3), and (4). This decrease shows that the structure becomes more stable and better integrated as more MoS_2_ is added.

**Figure 2:**
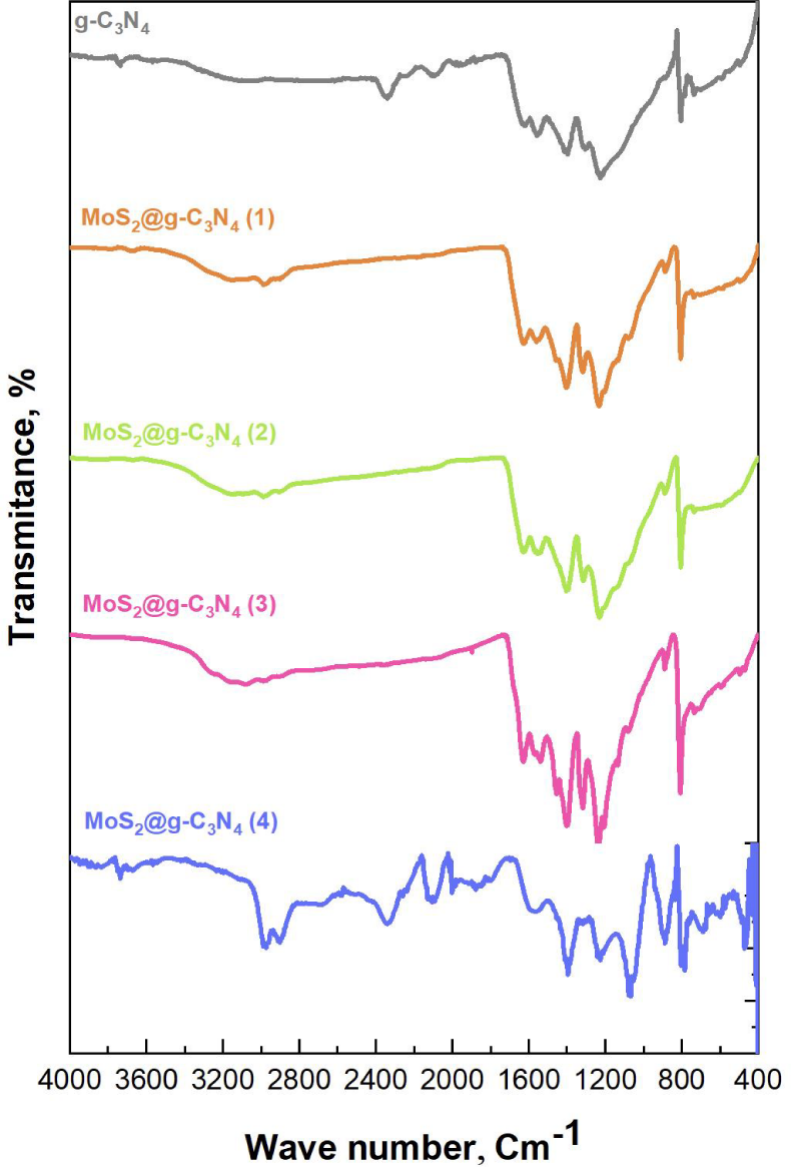
FT-IR spectra of the synthesized MoS_2_@ g-C_3_N_4_ nanocomposites.

Figure 2 illustrates the Fourier-transform infrared (FT-IR) spectra of g-C_3_N_4_ and MoS_2_@g-C_3_N_4_ nanocomposites synthesized with different compositions. The spectra confirm the presence of both g-C_3_N_4_ and MoS_2_ components within the nanomaterials, with noticeable changes as the MoS_2_ ratio increases. The structure of g-C_3_N_4_ is confirmed by a key peak at 811 cm^–1^, indicating the presence of its tri-s-triazine units. This peak confirms that the heptazine ring structure remains intact. Other important peaks in the 1200–1600 cm^–1^ range are related to C–N and C=N bonds, which are part of the g-C_3_N_4_ framework: 1231 cm^–1^: C–N stretching in the C–NH–C groups, 1315 cm^–1^: C–N stretching in the C–N(−C)–C group, 1406 cm^–1^: C–N stretching and 1534 cm^–1^: C=N stretching, representing the main aromatic backbone of g-C_3_N_4_.

The peak at 1624 cm^–1^, related to C=N bending, further confirms that the triazine rings are intact.^35,36^ The presence of MoS_2_ is evident from new peaks at 462 cm^–1^ and 591 cm^–1^, which correspond to Mo–S and S–S bonds, respectively.^37,38^ These peaks grow stronger as the MoS_2_ ratio increases (samples 1 to 4). As MoS_2_ content increases, some changes are observed in the g-C_3_N_4_ signals: the peaks at 1200–1600 cm^–1^ and the triazine breathing mode at 811 cm^–1^ become weaker. At the same time, the Mo–S bands become more prominent, highlighting the increasing presence of MoS_2_. The peak at 1534 cm^–1^ shifts slightly, indicating electronic interactions between g-C_3_N_4_ and MoS_2_. These interactions, along with the stronger Mo–S and S signals in MoS_2_@g-C_3_N_4_ (3) and MoS_2_@g-C_3_N_4_ (4), confirm the formation of a stable composite material.^39^

**Figure 3:**
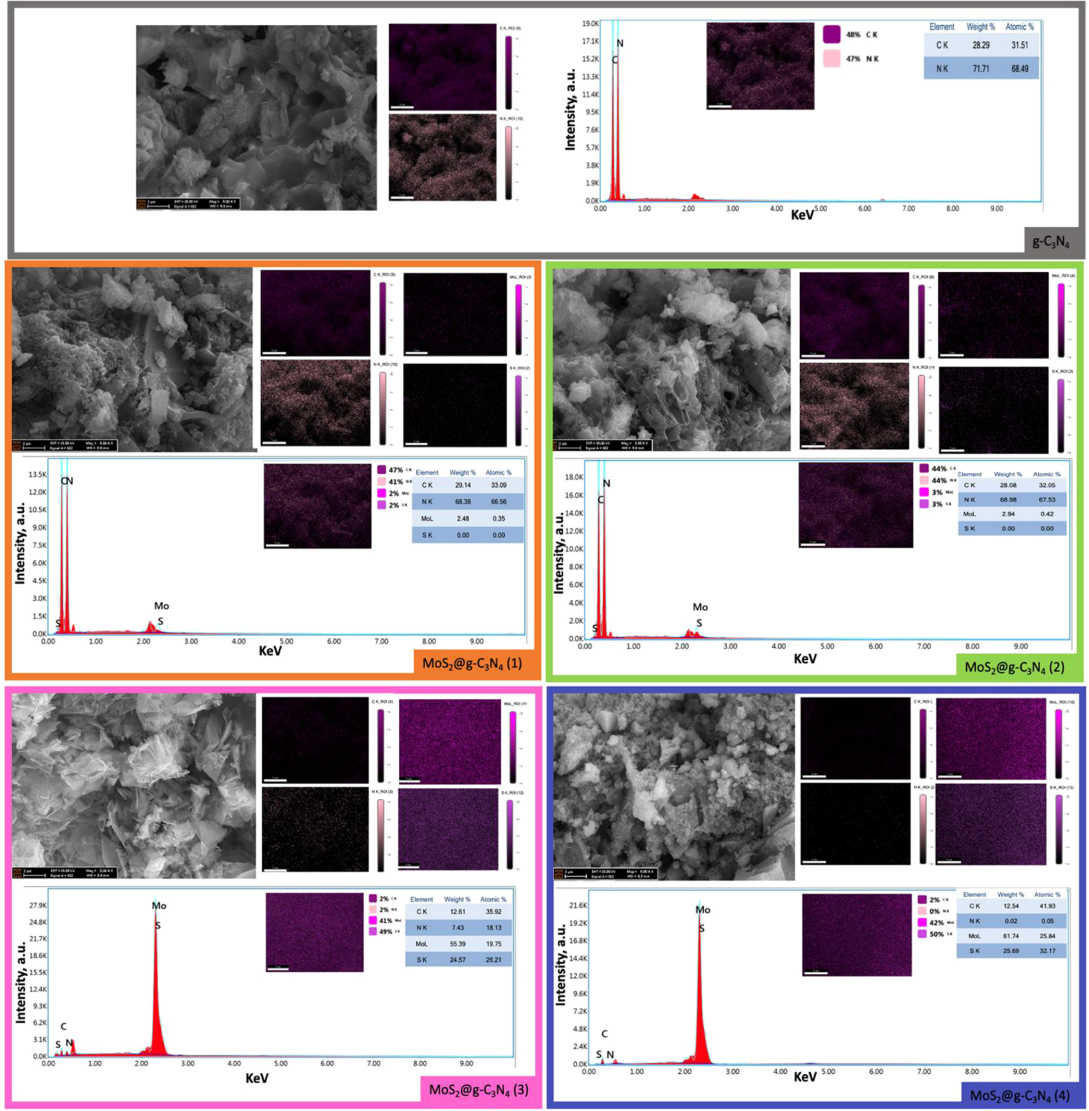
FESEM images, EDX, and elemental mapping of the of the MoS_2_@g-C_3_N_4_ nanocomposites.

FE-SEM was employed to acquire detailed morphological information for various synthesized nanocomposites. All images were captured at an accelerating voltage of 15.00 kV and a magnification of 50,000× (Figure 3). The FE-SEM images of the pure g-C_3_N_4_ exhibit a layered, sheet-like morphology with wrinkled surfaces and irregular pores typical of graphitic carbon nitride. MoS_2_@g-C_3_N_4_ nanocomposites with varying MoS_2_ NSs content reveal significant changes in surface morphology. For MoS_2_@g-C_3_N_4_ (1), the surface appears slightly rough with minimal flake-like structures, indicating a predominant g-C_3_N_4_ NSs matrix. As the ammonium molybdate content increases to 30 mg, more flake-like structures become apparent, suggesting a higher MoS_2_ NSs presence and rougher texture. The use of 300 mg of ammonium molybdate for the synthesis of MoS_2_@g-C_3_N_4_ nanocomposite exhibits a substantial increase in flake-like structures, with pronounced MoS_2_ adherence leading to a rougher and more textured surface. Lastly, 3000 mg of ammonium molybdate displays a densely packed surface with extensive flake-like structures, demonstrating a rough texture and significant MoS_2_ NSs coverage. This progression from smooth to highly textured surfaces with increasing MoS_2_ NSs content enhances surface area and adhesion properties, which are crucial for optimizing the material’s performance in various applications.^40,41^

The EDX spectra of the synthesized g-C_3_N_4_ and MoS_2_@g-C_3_N_4_ nanocomposites reveal clear compositional changes as MoS_2_ content increases. Pure g-C_3_N_4_ exhibits strong carbon (28.29 wt %) and nitrogen (71.71 wt %) peaks, with no molybdenum or sulfur detected, confirming its purity. In MoS_2_@g-C_3_N_4_ (1), the incorporation of Mo (2.28 wt %) and trace amounts of S is observed, while carbon and nitrogen remain dominant at 29.14 wt % and 68.38 wt%, respectively.

In MoS_2_@g-C_3_N_4_ (2), Mo increases slightly to 2.94 wt %, with trace sulfur detected, while carbon (28.08 wt %) and nitrogen (68.98 wt %) remain relatively high. Significant changes are evident in In MoS_2_@g-C_3_N_4_ (3), with Mo increasing to 55.39 wt % and S to 24.57 wt %, while carbon and nitrogen decrease to 12.61 and 7.43 wt %, respectively.

Finally, in MoS_2_@g-C_3_N_4_ (4), the highest Mo 61.74 wt % and S 24.89 wt % contents are observed, alongside drastically reduced carbon 12.54 wt % and nitrogen 0.92 wt %. These compositional trends, coupled with mapping data showing the homogeneous distribution of elements in all samples, confirm the systematic incorporation of MoS_2_ and its impact on the elemental composition of the nanocomposites.

The BET surface area and pore analysis reveal that increasing MoS_2_ content significantly enhances the textural properties of MoS_2_@g-C_3_N_4_ nanocomposites. For MoS_2_@g-C_3_N_4_ (1), the BET surface area was 10.38 m^2^/g, with an external surface area of 9.00 m^2^/g and a total pore volume of 0.0597 cm^3^/g. The average pore width was 230.16 Å (BET) and 268.47 Å (BJH). In contrast, MoS_2_@g-C_3_N_4_ (3) exhibited a much larger BET surface area of 69.53 m^2^/g, with an external surface area of 69.33 m^2^/g and a significantly higher pore volume of 0.4193 cm^3^/g. Its average pore width was 241.19 Å (BET) and 214.08 Å (BJH). Comparing both composites, MoS_2_@g-C_3_N_4_ (3) has a surface area about 6.7 times larger and a pore volume about 7 times higher than MoS_2_@g-C_3_N_4_ (1), with similar pore widths. This suggests that MoS_2_@g-C_3_N_4_ (3) possesses a more porous structure, which may improve its performance in applications requiring high surface area and porosity, such as adsorption.

### 3.2 Optimization of MoS_2_ Ratios in MoS_2_@g-C_3_N_4_ Nanocomposite

**Figure 4:**
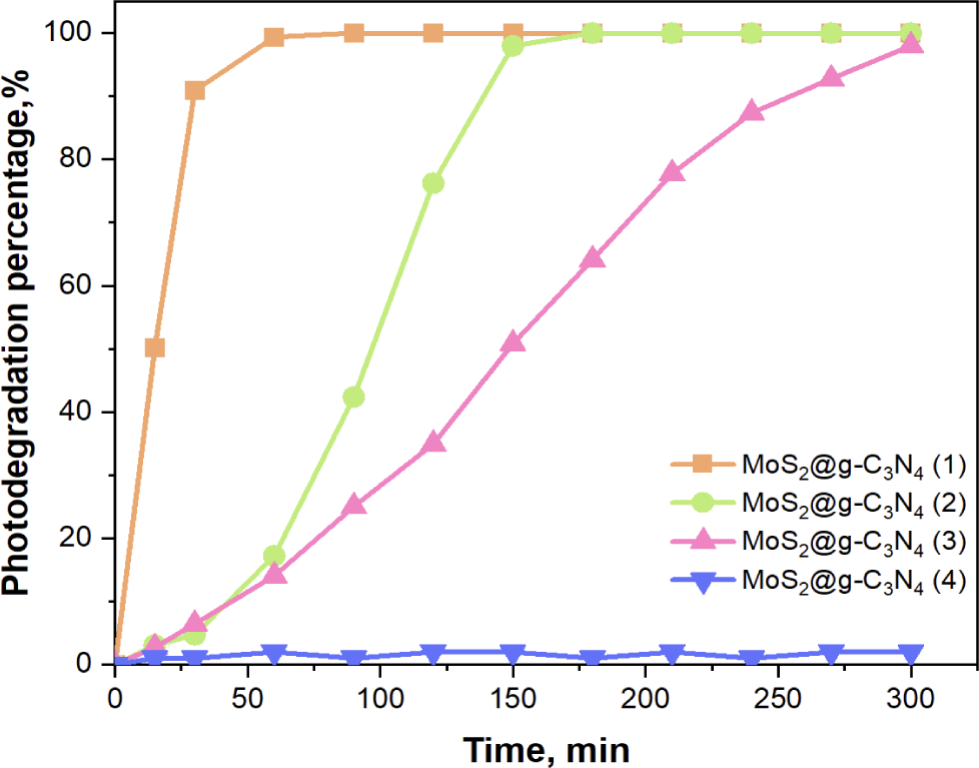
Effect of different MoS_2_ compositions in MoS_2_@g-C_3_N_4_ nanocomposites on the photocatalytic removal of RhB.

The effect of changing the amount of ammonium molybdate used in forming MoS_2_ NSs on the photocatalytic performance efficiency of MoS_2_@g-C_3_N_4_ nanocomposite was investigated on RhB degradation. For this purpose, 25 mg each of MoS_2_@g-C_3_N_4_ (1), MoS_2_@g-C_3_N_4_ (2), MoS_2_@g-C_3_N_4_ (3) and MoS_2_@g-C_3_N_4_ (4) nanocomposites were used for photocatalytic experiments. The results of RhB photodegradation obtained using the different nanocomposites are presented in Figure 4. According to the results, MoS_2_@g-C_3_N_4_ (1) nanocomposite exhibited the highest photocatalytic efficiency, achieving complete RhB degradation within 60 min. MoS_2_@g-C_3_N_4_ (2) and MoS_2_@g-C_3_N_4_ (3) nanocomposites showed progressively longer degradation times of 120 and 300 min to achieve the same level of degradation. MoS_2_@g-C_3_N_4_ (4) nanocomposite did not exhibit any photocatalytic activity, likely due to the complete coverage of g-C_3_N_4_ NSs surface by MoS_2_ NSs, which hindered its catalytic function. These results underscore the significant influence of catalyst composition and structure on photocatalytic performance.

**Figure 5:**
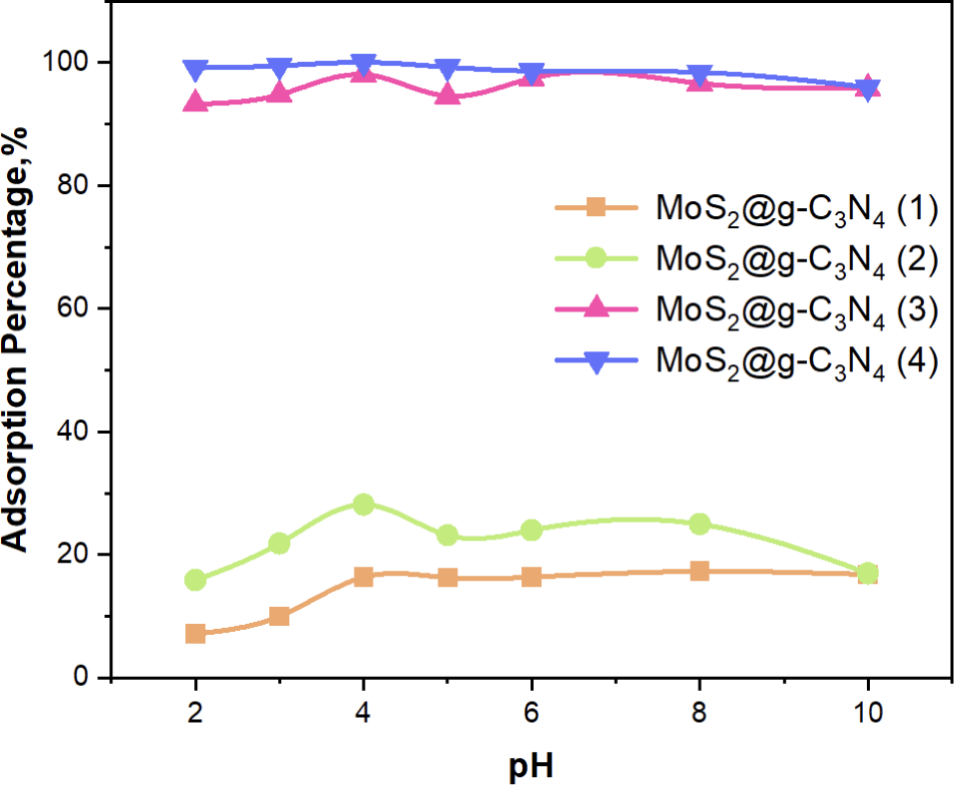
Effect of different MoS_2_ compositions in MoS_2_@g-C_3_N_4_ nanocomposites on the adsorption-based removal of RhB (*n* = 3).

To investigate the influence of different amounts of ammonium molybdate used in the formation of MoS_2_ NSs on the adsorption performance efficiency of MoS_2_@g-C_3_N_4_ nanocomposites were conducted with pH levels ranging from 2.0 to 10.0 on RhB. In adsorption-based removal studies, the pH of the solution housing the analyte is a significant determinant of adsorption efficiency. This influence arises from the pH’s ability to alter the molecular state of the target compounds, whether they exist in an ionic or neutral form. The results from Figure 5 highlight the pH-dependent adsorption behavior of RhB on MoS_2_@g-C_3_N_4_ nanocomposite. At pH 4.0, all synthesized MoS_2_@g-C_3_N_4_ variants demonstrated their highest adsorption efficiencies: MoS_2_@g-C_3_N_4_ (4) and MoS_2_@g-C_3_N_4_ (3) nanocomposites achieved >98% adsorption, indicating their strong affinity for RhB molecules under these conditions. In contrast, MoS_2_@g-C_3_N_4_ (2) and MoS_2_@g-C_3_N_4_ (1) nanocomposites exhibited lower adsorption efficiencies of 28% and 16%, respectively. This trend can be attributed to the inherent adsorption properties of MoS_2_, which possesses a layered structure with abundant active sites that facilitate effective interactions with adsorbates; it is important to note that g-C_3_N_4_ primarily serves as a photocatalyst rather than an adsorbent.^42,43^ When the results obtained are examined, it is seen that all adsorption efficiencies increase up to pH 4.0, and adsorption efficiencies decrease after this pH value. The possible adsorption mechanism can be explained as follows. The p*K*_a_ value of RhB is 4.2. Above pH 4.0, the negatively charged adsorbent repels the negatively charged RhB molecule, and as the pH value increases, the repulsion force increases. In other words, electrostatic interaction forces between RhB and adsorbents are effective in adsorption.

When the experimental results related to both photocatalytic and adsorption-based removal of RhB were examined, it was determined that MoS_2_@g-C_3_N_4_ (1) and MoS_2_@g-C_3_N_4_ (2) nanocomposites could not be used in adsorption-based removal studies due to their low adsorption properties. However, it was understood that MoS_2_@g-C_3_N_4_ (4) nanocomposite could not be used in photocatalytic removal studies due to its lack of photocatalytic properties. As a result of all experimental results, it was seen that MoS_2_@g-C_3_N_4_ (3) nanocomposite was the optimum material that could be used in both removal methods.

### 3.3 Application of the Optimized Multifunctional Material for the Removal of SLD, FLX, and RhB

#### 3.3.1 Photocatalytic Degradation-Based Removal Experiments

The photocatalysis-based removal experiments were conducted in two stages to assess the photodegradation of SLD, FLX, and RhB: first without the photocatalyst and then in its presence.

In the initial stage, the prepared 20 ppm of SLD, FLX, and RhB solutions were placed under a 400 W UV lamp 15 cm above the solution’s surface. The systems were exposed to UV irradiation for a total duration of 300 min. To monitor the extent of photodegradation, 1.0 mL samples were withdrawn from the solutions at 30 min intervals. Each sample was analyzed using Ultra Performance Liquid Chromatography with Diode-Array Detection (UPLC-DAD) to determine the concentrations of SLD and FLX, while a UV–vis spectrophotometer was used to assess the remaining RhB in the solution.

The same experimental stages were repeated in the second stage with an appropriate amount of MoS_2_@g-C_3_N_4_ (3) nanocomposite. After the photocatalytic degradation process, analyses were carried out using the UPLC-DAD and UV–vis spectrophotometer.

**Figure 6:**
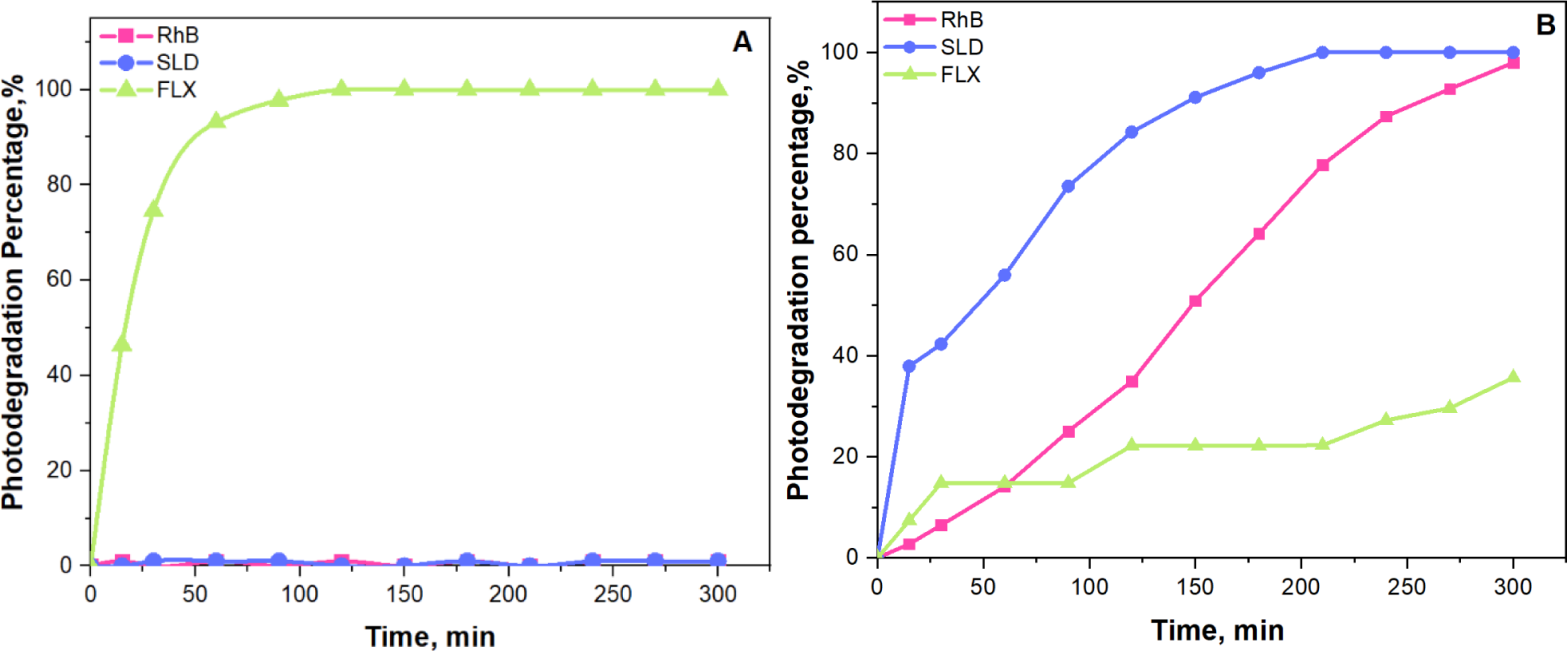
Photocatalytic degradation-based removal of RhB, SLD, and FLX (A) without photocatalyst, (B)in the presence of the MoS_2_@g-C_3_N_4_ (3) photocatalyst.

The results of the photocatalysis-based degradation of RhB, SLD, and FLX with and without a photocatalyst are represented in Figure 6. According to these results, FLX degrades without using a photocatalyst, with 100% degradation achieved within 120 min. This indicates that FLX is highly susceptible to UV rays without a photocatalyst; this can be attributed to differences in their degradation mechanisms. FLX likely undergoes direct UV-induced cleavage of its ether bond in photolysis, resulting in rapid degradation into trifluoromethylphenol (TFMP) and fluoride ions. In contrast, photocatalysis relies heavily on the surface interaction of FLX with the catalyst and the generation of electron–hole pairs and reactive species, such as hydroxyl radicals, under UV irradiation. These reactive species can degrade FLX, but the process appears less efficient because of the probable formation of stable intermediates that are more selective than FLX. These intermediates may adsorb onto the catalyst surface, hindering further FLX degradation. Other substances in the solution could compete for reactive species and further reduce FLX’s degradation efficiency.^44,45^

RhB and SLD showed significant stability toward UV rays, with minimal degradation observed without a photocatalyst. However, using a photocatalyst significantly enhanced the degradation rates of both compounds. Specifically, 99% of RhB was degraded within 300 min, and 100% of SLD was degraded within 210 min. Highlighting the enhanced efficiency of photocatalysis in degrading these compounds compared to UV irradiation alone. These findings underscore the effectiveness of MoS_2_@g-C_3_N_4_ (3) nanocomposite as a photocatalyst in environmental remediation processes involving RhB, SLD, and FLX.

**Figure 7:**
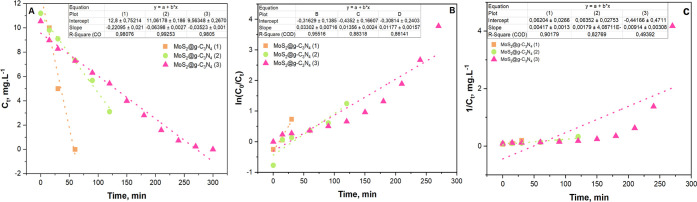
Kinetic plot for RhB photodegradation using the synthesized MoS_2_@g-C_3_N_4_ nanocomposites. (A) Zero-order model, (B) first-order model, and (C) second-order model.

Figure 7 illustrates the kinetics models applied to RhB photodegradation using the synthesized MoS_2_@g-C_3_N_4_ nanocomposites, including the pseudozero-order, first-order, and second-order models. Table 2 summarizes the rate constants (*K*) and correlation coefficients (*R*^2^). Notably, the pseudozero-order model shows a high correlation coefficient (*R*^2^ ≥ 0.98), meaning the degradation rate remains constant as long as the photocatalyst is actively exposed to light. This finding indicates a stable and predictable reaction rate, emphasizing the reliability of the photocatalytic process.

**Table 2:** Kinetic Parameters of the Photodegradation of RhB Using the Synthesized MoS_2_@g-C_3_N_4_ Nanocomposites

	MoS_2_@g-C_3_N_4_ (1)		MoS_2_@g-C_3_N_4_ (2)		MoS_2_@g-C_3_N_4_ (3)	
kinetic model	rate constant *K*	*R*^2^	rate constant *K*	*R*^2^	rate constant *K*	*R*^2^
pseudozero-order	0.22 (mg·L^–1^·min^–1^)	0.98	0.06 ( mg·L^–1^·min^–1^)	0.99	0.04 (mg·L^–1^·min^–1^)	0.98
pseudo-first-order	0.03 (min^–1^)	0.95	0.01 (min^–1^)	0.88	0.01 (min^–1^)	0.86
pseudo-second-order	0.004 (L·mg^–1^·min^–1^)	0.90	0.002 (L·mg^–1^·min^–1^)	0.82	0.001 (L·mg^–1^·min^–1^)	0.49

**Figure 8:**
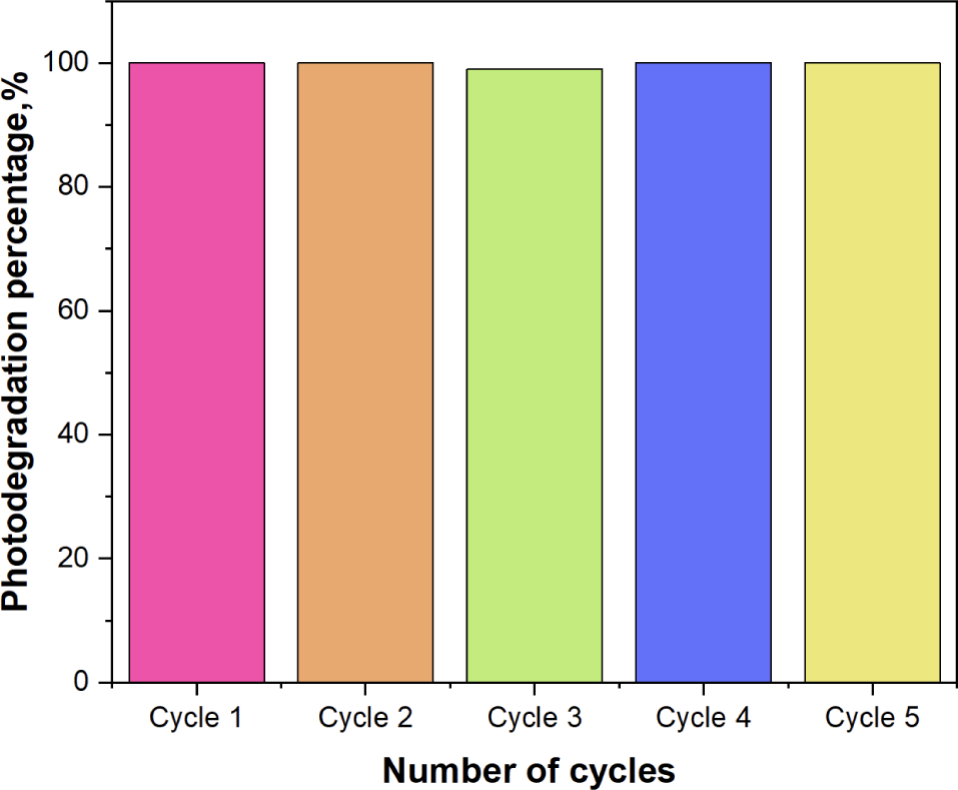
Reusability cycles of MoS_2_@g-C_3_N_4_ (3) nanocomposite for the RhB photodegradation process.

The reusability of the photocatalyst MoS_2_@g-C_3_N_4_ (3) nanocomposite was tested over five experimental cycles, each lasting 300 min. As shown in Figure 8, the material maintained an efficiency of 99% to 100% throughout the five cycles, demonstrating that it can be effectively reused for RhB photodegradation up to five times.

#### 3.3.2 Adsorption-Based Removal Experiments

To investigate the influence of solution pH on the adsorption-based removal of RhB, SLD, and FLX, experiments were conducted using MoS_2_@g-C_3_N_4_ (3) nanocomposite as adsorbent at different pH levels ranging from 2.0 to 10.0. Each pH level was simulated by preparing 10 mL of solutions containing 10 mg·L^–1^ of SLD, 5 mg·L^–1^ of FLX, and 10 mg·L^–1^ of RhB, respectively, using suitable buffer solutions. A fixed amount of 25 mg of adsorbent was added to each solution. The mixtures were thoroughly mixed using a vortex device for 20 min, followed by 10 min of ultrasonic treatment to enhance adsorption. After adsorption, the mixtures were centrifuged to separate the sorbent from the solution, and 1 mL samples were extracted for pollutant concentration determination.

**Figure 9:**
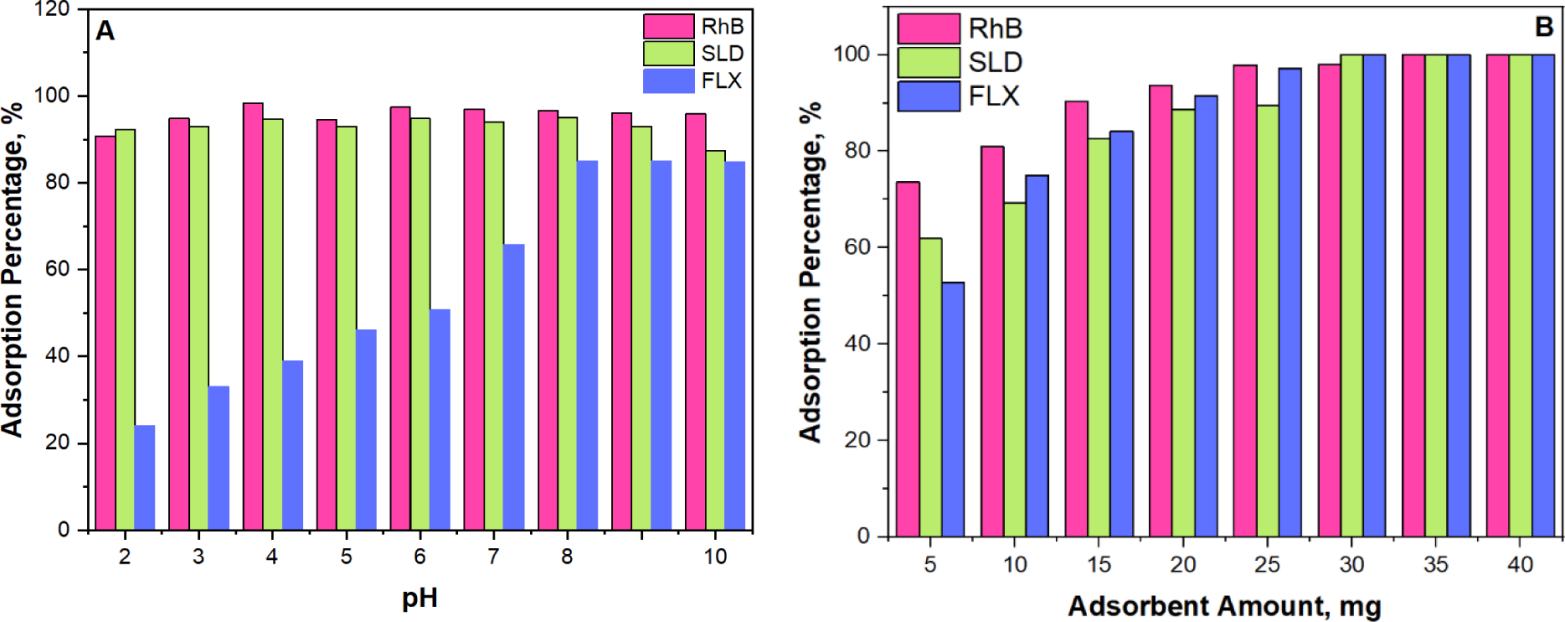
(A) Effect of the pH of the solution and (B) effect of the amount of adsorbent for efficiency of adsorption-based removal of RhB, SLD, and FLX.

**Figure 10:**
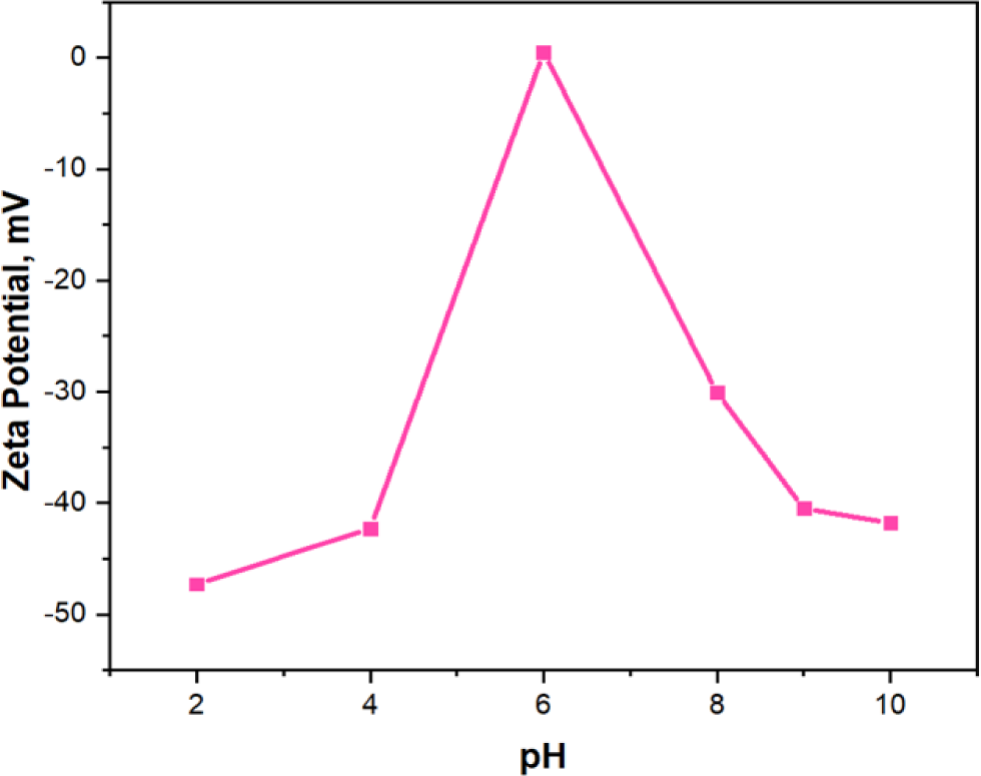
Zeta potential of the synthesized MoS_2_@g-C_3_N_4_ (3) as function of pH.

RhB concentrations were evaluated using a UV–visible spectrophotometer, while SLD and FLX concentrations were assessed using a UPLC-DAD device. Figure 9A illustrates the variation of RhB, SLD and FLX adsorption percentages as a function of solution pH. The highest adsorption percentages were 96% at pH 4.0 for RhB, 96% at pH 8.0 for SLD, and 85% at pH 9.0 for FLX, respectively. These outcomes can be explained by the interplay between the charge states of the molecules and the adsorbent surface, influenced by their respective p*K*_a_ values and the solution pH. At pH 4.0, RhB is positively charged. While the adsorbent surface is negatively charged (as confirmed by the zeta potential in Figure 10, between pH 2–6 and pH 6–10, the adsorbent is negatively charged), leading to strong electrostatic attraction and maximum adsorption efficiency.^46^ SLD’s partially deprotonated state at pH 8.0, comprising a mixture of positively charged and neutral forms, facilitates electrostatic interaction with the negatively charged adsorbent surface, resulting in optimal adsorption. However, above pH 9, SLD becomes predominantly neutral, reducing its interaction with the adsorbent and decreasing adsorption efficiency.^47^ Similarly, FLX, with a p*K*_a_ of 9.8, is mostly protonated and positively charged at pH 9.0, promoting strong electrostatic attraction with the negatively charged surface. Beyond this pH, protonation and surface charge changes diminish the adsorption efficiency.^48^

To optimize the adsorption-based removal conditions of RhB, SLD, and FLX, the amount of adsorbent was evaluated using the same experimental setup previously described. The pH levels were set to 4.0 for RhB, 8.0 for SLD, and 9.0 for FLX, with the amount of the photocatalyst varied from 5 mg to 40 mg. The results of this experiment are presented in Figure 9B. According to these results, increasing MoS_2_@g-C_3_N_4_ (3) nanocomposite quantity led to a proportional increase in adsorption percentages, reaching a complete removal efficiency of 100%. Specifically, 35 mg of adsorbent proved optimal for RhB removal, while 30 mg was optimal for removing SLD and FLX. These findings underscore the effective utilization of MoS_2_@g-C_3_N_4_ (3) nanocomposite in maximizing adsorption capacity for different pollutants, highlighting tailored adsorbent dosages as critical for efficient environmental remediation strategies.

**Figure 11:**
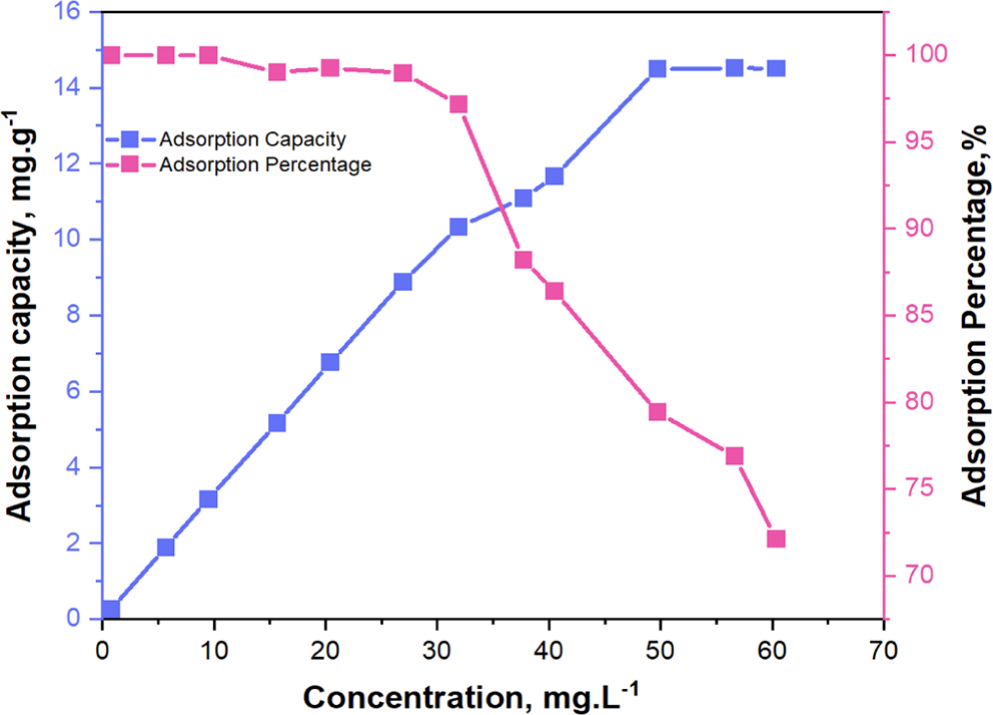
Adsorption capacity and adsorption percentage of MoS_2_@g-C_3_N_4_ (3) as a function of RhB concentration (*n* = 3).

The adsorption capacity and isotherm of the optimized nanomaterial were evaluated with different initial concentrations of RhB, as shown in Figure 11. The adsorption capacity (Q_t_) ranged from 0.262 mg·g^–1^ to 14.517 mg·g^–1^, reaching a maximum that did not exceed 14.517 mg·g^–1^. This indicates that the adsorbent’s ability to remove RhB varies with concentration but reaches a saturation point where the material cannot adsorb additional RhB, suggesting full occupation of the active sites at higher concentrations. The adsorption percentage ranged from 72% to 100%, showing the efficiency of the adsorbent in different concentrations.

**Figure 12:**
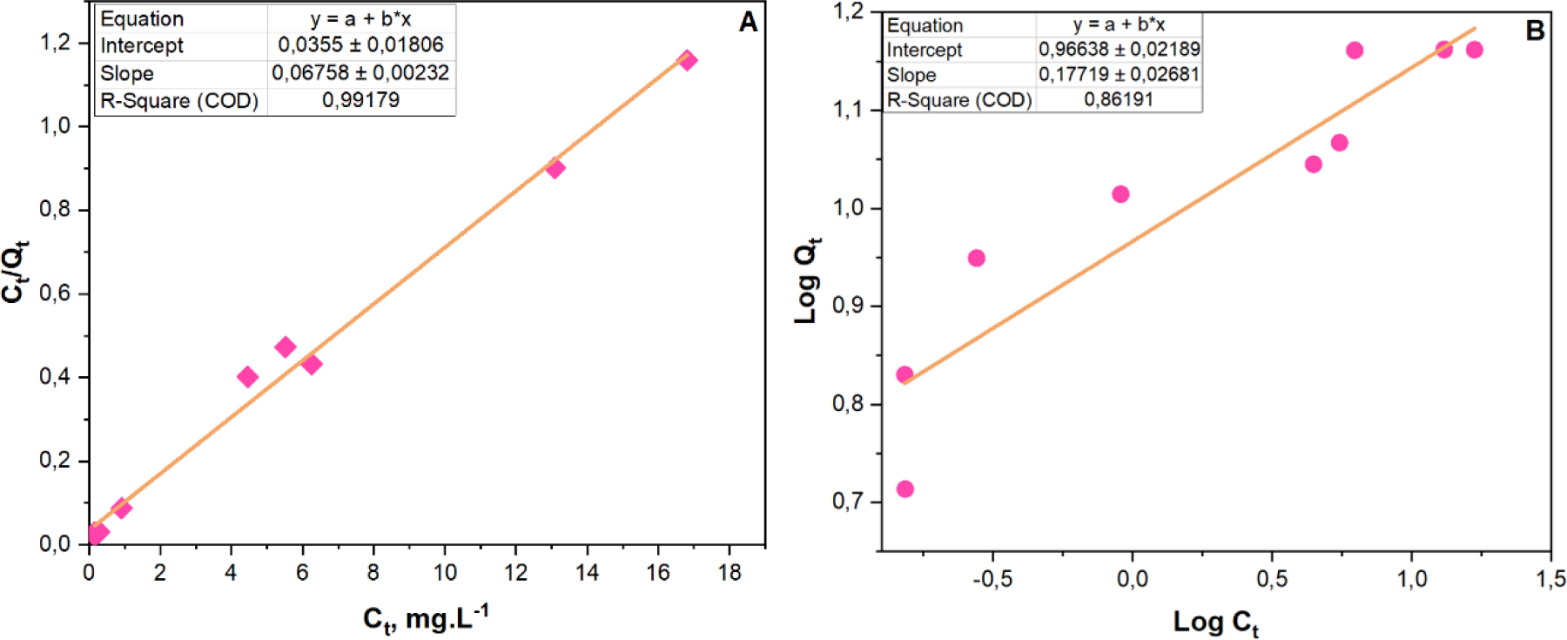
RhB’s adsorption isotherms. (A) Langmuir and (B) Freundlich (*n* = 3).

Figure 12 and Table 3 show the results of the isotherm study using the Langmuir isotherm model A and the Freundlich isotherm model B. The results stipulate that the Langmuir model provided an excellent fit to the data (*R*^2^ = 0.992), indicating monolayer adsorption on a homogeneous surface with a maximum adsorption capacity of 14.798 mg·g^–1^ and a Langmuir constant *K*_L_ of 1.904 L·mg^–1^.

**Table 3:** Langmuir and Freundlich Isotherm Constants of RhB on MoS_2_@g-C_3_N_4_ (3) Nanocomposite

Langmuir isotherm model			Freundlich isotherm model		
*R*^2^	*Q*_E_	*K*_L_	*R*^2^	*K*_f_	*n*
0.992	14.798	1.904	0.862	9.255	5.644

## 4 Conclusions

This study successfully synthesized and optimized a multifunctional nanomaterial based on MoS_2_ and g-C_3_N_4_, demonstrating its high efficiency in removing Rhodamine B (RhB), Sildenafil citrate (SLD), and Fluoxetine (FLX) from aqueous solutions. Four distinct ratios of MoS_2_ to g-C_3_N_4_ nanocomposites were synthesized and characterized using FT-IR, XRD, and SEM techniques, with the MoS_2_@g-C_3_N_4_ (3) nanocomposite emerging as the most effective material due to its exceptional adsorption and photocatalytic properties. Adsorption studies revealed that the material exhibited pH-dependent behavior, achieving maximum removal efficiencies of 96% for RhB at pH 4.0, 96% for SLD at pH 8.0, and 85% for FLX at pH 9.0. The photocatalytic degradation experiments further highlighted the enhanced performance of MoS_2_@g-C_3_N_4_ (3), which achieved complete degradation of RhB and SLD under UV irradiation within 300 and 210 min, following pseudozero-order kinetics.

The nanocomposite also demonstrated excellent reusability, maintaining its efficiency across five consecutive photocatalytic cycles, showcasing its stability and potential for long-term applications in water treatment. Its multifunctional nature, combining robust adsorption capabilities with efficient photocatalytic activity, positions it as a versatile material capable of addressing diverse pollutants through complementary mechanisms.
